# SuroTex: Surrounding texture dataset

**DOI:** 10.1016/j.dib.2025.111292

**Published:** 2025-01-10

**Authors:** Muhammad Ardi Putra, Wahyono, Agus Harjoko

**Affiliations:** Department of Computer Science and Electronics, Universitas Gadjah Mada, Indonesia

**Keywords:** Computer Vision, Image processing, Feature extraction, Image classification, Material classification

## Abstract

Texture analysis can be considered as one of the most important topics in the field of image processing and computer vision. However, the existing texture datasets such as KTH-TIPS, KTH-TIPS2, USPTex, DTD, and ALOT still have limitations which causes the resulting analysis on different texture classification algorithms to be somewhat unreliable. The two main reasons behind this problem are the limited number of texture classes and the non-uniformity of the image sizes. To address this issue, we introduce a new texture classification dataset named SuroTex (Surrounding Texture) Dataset. The dataset, which was collected back in November 2023 in Ulsan, South Korea, contains 5000 image textures. These number of samples are divided into 50 classes, each with 100 RGB samples of size 400 × 400 pixels. Future researchers can use SuroTex for a benchmark dataset both for general image classification or specifically for texture classification tasks. Additionally, models trained on this dataset can also act as a pre-trained model which can further be fine-tuned for similar purposes such as texture-based material classification.

Specifications Table

**Table:** 

Subject	Computer Vision and Pattern Recognition.
Specific subject area	Image classification, image processing
Data format	Raw
Type of data	Image
Data collection	Images were captured using Samsung Galaxy Note 10 smartphone camera with the resolution of 2268 × 4032 (9.1 megapixels). Any textured surfaces from both indoor and outdoor environment were included. Python OpenCV was then utilized to perform resizing and cropping to form the final 400 × 400 image.
Data source location	Ulsan, South Korea
Data accessibility	Repository name: Mendeley Data Data identification number: 10.17632/t6mdbpy2yk Direct URL to data: https://data.mendeley.com/datasets/t6mdbpy2yk/
Related research article	None

## 1 Value of the Data

• The SuroTex Dataset provides a collection of texture images from various surfaces that can commonly be found in daily life. The dataset, which contains up to 5000 images from 50 classes, presents a new challenge for researchers in the field of image processing and computer vision to conduct experiments on texture classification and analysis.
• This dataset can help researchers in developing texture feature extraction algorithms, in a sense that it can be used as a benchmark dataset to validate the performance of a specific texture classification method. The SuroTex Dataset can be used for this purpose alongside with the existing texture classification datasets, such as KTH-TIPS (Kungliga Tekniska högskolan—Textures under varying Illumination, Pose and Scale) [1], KTH-TIPS2 [2], USPTex [3], DTD (Describable Texture Dataset) [4], and ALOT (Amsterdam Library of Textures) [5].
• The model trained with SuroTex Dataset can be perceived as a pre-trained model. This model can further be fine-tuned or transfer-learned to match future research needs, especially when the actual classification task also relies on texture features, such as material classification, fabric analysis, or satellite imagery-based terrain classification.

## 2 Background

Texture feature extraction is one of the most important aspects in the field of image processing and computer vision. In order to develop texture feature extraction algorithms, it is necessary to employ a high-quality texture classification dataset to validate whether the algorithm to be developed really makes improvements over the existing ones. Unfortunately, many existing texture datasets do not completely fulfill this requirement. The KTH-TIPS [1] and KTH-TIPS2 [2] datasets only have limited number of texture classes. The USPTex dataset [3] can actually be used to overcome this problem as it contains much larger number of classes. However, the number of samples in each class in this dataset is considerably small. Despite solving the issues regarding the amount of data, the image sizes in the DTD [4] and ALOT [5] datasets are not uniform. This requires researchers to either resize or crop the images, which leads to changes in the textural structures. Due to the above reasons, we propose a new texture dataset that contains a wide variety of classes and samples, while at the same time having uniform image dimensions, allowing SuroTex to be able to be used as a reliable benchmark dataset.

## 3 Data Description

**Fig. 1:**
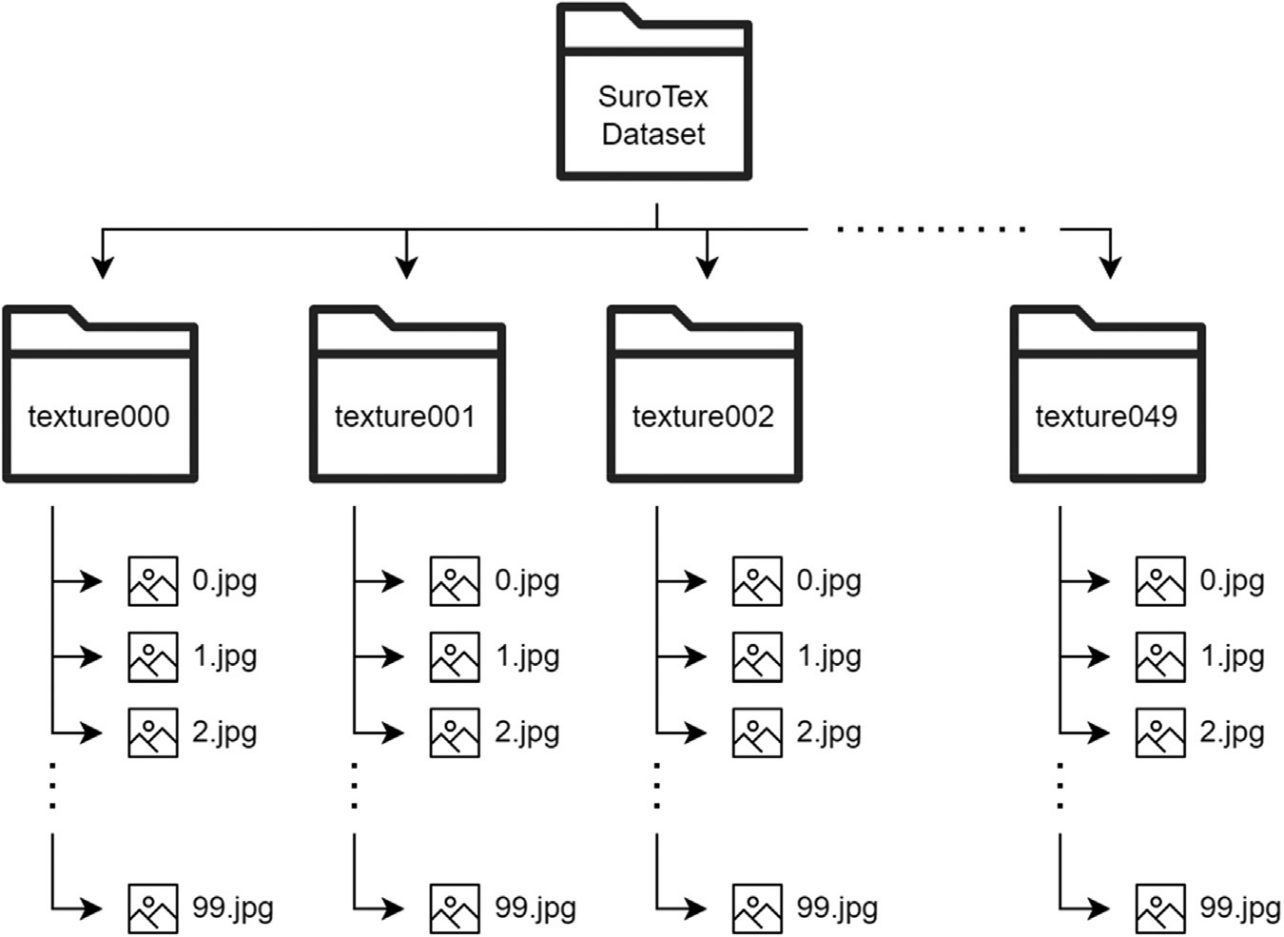
The directory structure of the SuroTex dataset.

**Fig. 2:**
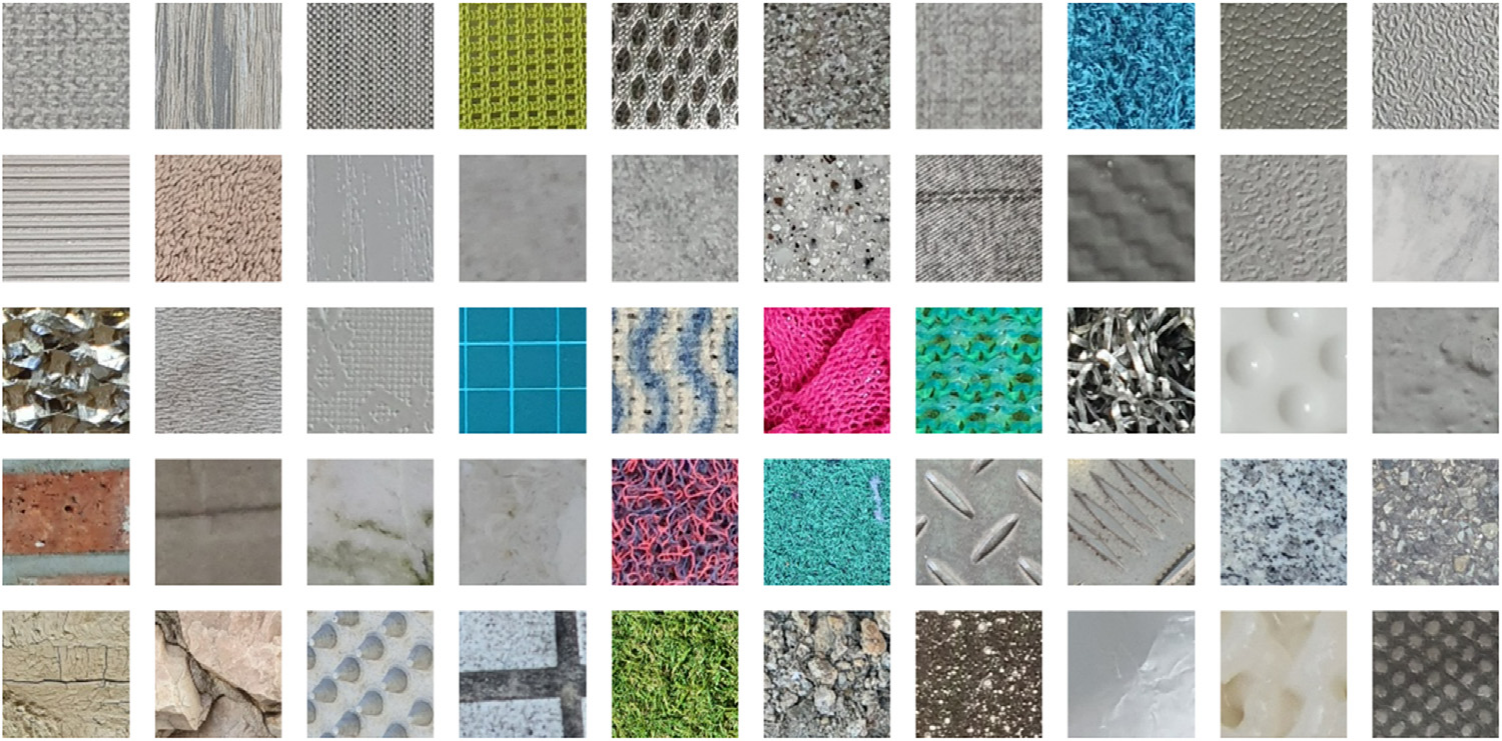
Examples of texture images in the SuroTex dataset from different classes (starting from texture000 at the top-left, texture001 next to it, all the way to texture049 at the bottom-right).

**Fig. 3:**
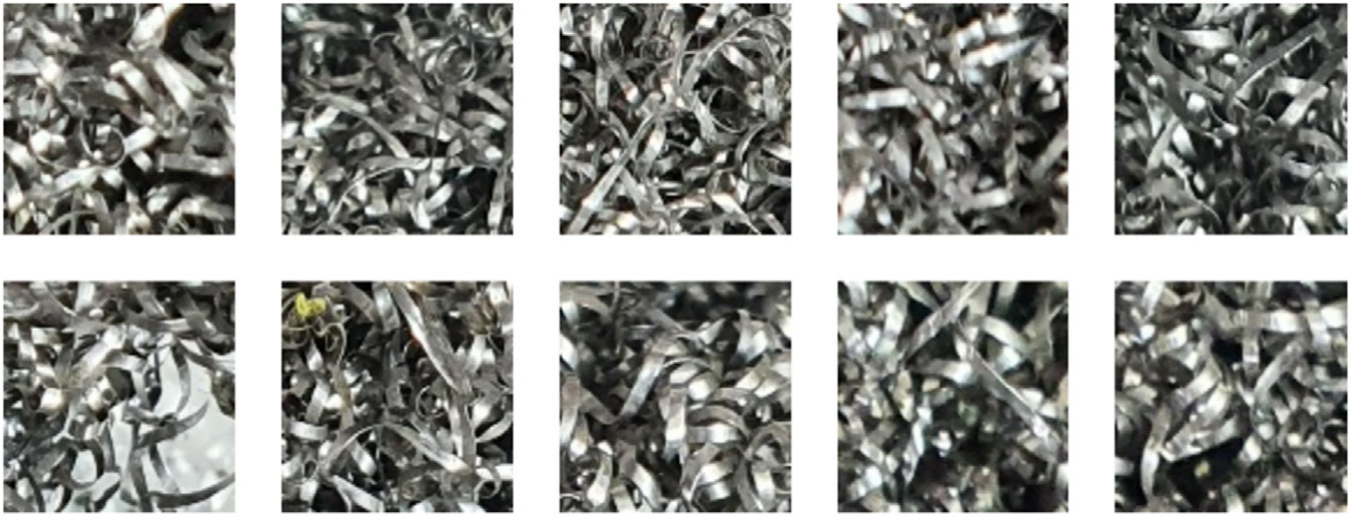
Several images from texture027 class.

The SuroTex Dataset comprises 5000 texture images evenly distributed across 50 classes. These images are stored in separate folders according to their class, namely texture000, texture001, texture002, up to texture049. Note that the texture names are not explicitly provided because these images are captured arbitrarily from the surroundings, where the detailed information regarding the specific materials is unavailable. In fact, there are many instances where the materials across different classes are similar, and the differences between them are primarily the repeating patterns rather than the material composition itself. Every single of these folders contains 100 RGB images with the resolution of 400 × 400 pixels, which the image files are named with integer from 0.jpg to 99.jpg. The size of these images is larger than those found in the KTH-TIPS, KTH-TIPS2, USPTex and ALOT datasets, making it more suitable for training state-of-the-art models like ViT (Vision Transformer) or CNN-based architectures which typically require an input size of 224 × 224. The large number of images in this dataset and its uniform image dimensions may reduce the need for data augmentation and preprocessing prior to being used for training a specific model. These features address the limitations of the existing datasets, which usually have either smaller number of samples or inconsistent image dimensions. The details of this directory structure can be seen in Fig. 1. Meanwhile, Fig. 2 shows an image from each of the 50 classes, while Fig. 3 displays several images from the same class.

It is necessary to acknowledge that the color distribution of the entire dataset is not entirely uniform, in which most textures in this dataset have grayish colors. However, color information is typically discarded when it comes to classifying textures. In this case the authors provide the raw RGB file so that researchers can have a better visualization of what the actual texture looks like.

## 4 Experimental Design, Materials and Methods

The texture images in the proposed SuroTex Dataset were captured in Ulsan, South Korea in November 2023. This dataset contains textures from various surfaces commonly found in daily life, including both the ones from indoor and outdoor areas such as objects made of different materials, grass, walls, or fabrics. The illumination within the dataset varies due to differences in surface locations. Despite these varying conditions, the textures contained in the image are free from shadow boundaries and are clearly recognizable by human eyes.

**Fig. 4:**
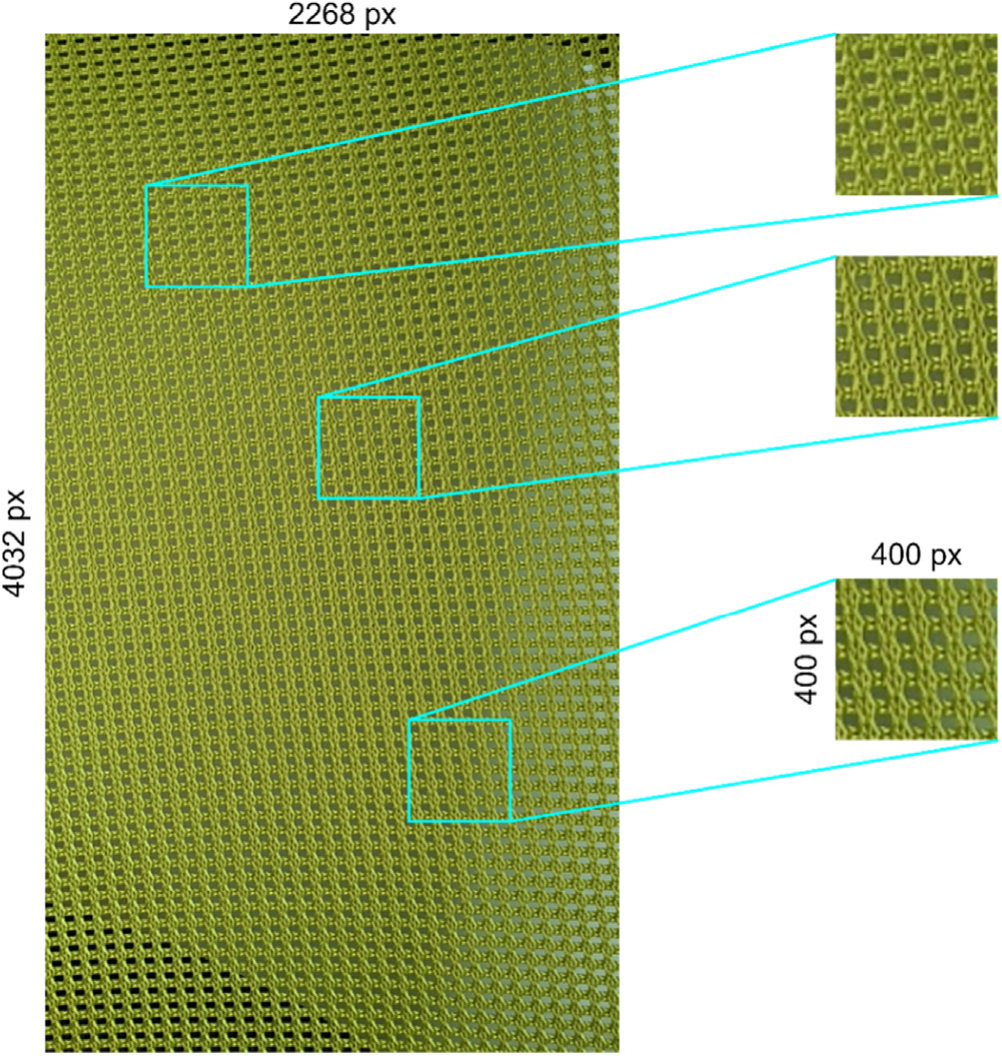
An example of how the 400×400 texture images are cropped from the raw image (image from texture003 class).

All images were captured using the exact same camera, namely the Samsung Galaxy Note 10 smartphone with the resolution of 2268 × 4032 (9.1 megapixels). This smartphone was configured to automatically convert the captured image into JPG format. The first data collection step was to capture 5 images from the same surface for each class. Neither color editing nor grayscaling applied to these images. All these images were then cropped at random locations 20 times, each with the cropping size of 400 × 400 pixels (Fig. 4). Finally, the resulting images are stored in the file structure previously displayed in Fig. 1.

Talking about the image file names, they are all named with a number starting from 0.jpg all the way to 99.jpg. In order to make the directory structure simpler, these image files are not grouped into its corresponding original image source. However, the cropped images are organized such that every set of 20 consecutive images are derived from the same original image. For example, 0.jpg to 19.jpg are from the first original image, 20.jpg to 39.jpg are from the second original image, and so on. With this information, it is possible to use this dataset to perform 5-fold cross validation method where the testing samples are taken from a different original image.

## Limitations

Not applicable.

## Ethics Statement

The authors have read and follow the ethical requirements for publication in Data in Brief. This dataset does not involve human subjects, animal experiments or data collected from social media platforms.

## CRediT authorship contribution statement

**Muhammad Ardi Putra:** Methodology, Data curation, Writing – original draft. **Wahyono:** Conceptualization, Supervision, Writing – review & editing. **Agus Harjoko:** Supervision, Project administration, Writing – review & editing.

## Data Availability

Mendeley DataSuroTex: Surrounding Texture Dataset (Original data).
